# Immobilization of myoglobin on Au nanoparticle-decorated carbon nanotube/polytyramine composite as a mediator-free H_2_O_2_ and nitrite biosensor

**DOI:** 10.1038/srep18390

**Published:** 2015-12-17

**Authors:** A. T. Ezhil Vilian, Vediyappan Veeramani, Shen-Ming Chen, Rajesh Madhu, Cheol Hwan Kwak, Yun Suk Huh, Young-Kyu Han

**Affiliations:** 1 Electroanalysis and Bioelectrochemistry Lab, Department of Chemical Engineering and Biotechnology, National Taipei University of Technology, No. 1, Section 3, Chung-Hsiao East Road, Taipei 106, Taiwan, ROC; 2 Department of Biological Engineering, Biohybrid Systems Research Center (BSRC), Inha University, Incheon 402-751, Republic of Korea; 3 Department of Energy and Materials Engineering, Dongguk University-Seoul, Seoul 100-715, Republic of Korea

## Abstract

A novel composite film was designed for use as a highly selective mediator-free amperometric biosensor, and a method was created for accomplishing direct electrochemistry of myoglobin on a multi-walled carbon nanotube and tyramine-modified composite decorated with Au nanoparticles on a glassy carbon electrode. The ultraviolet-visible and electrochemical impedance spectroscopy results showed that myoglobin retained its native conformation in the interaction with Au-PTy-*f*-MWCNT. The surface coverage of Mb-heme-Fe^(II)/(III)^ immobilized on Au-PTy-*f*-MWCNT and the heterogeneous electron-transfer rate constant were 2.12 × 10^−9^ mol cm^−2^ and 4.86 s^−1^, respectively, indicating a higher loading capacity of the nanocomposite for direct electron transfer of Mb onto the electrode surface. The proposed Mb/Au-PTy-*f*-MWCNT biofilm exhibited excellent electrocatalytic behavior toward the reduction of H_2_O_2_ and the oxidation of nitrite with linear ranges of 2 to 5000 μM and 1 to 8000 μM and lower detection limits of 0.01 μM and 0.002 μM, respectively. An apparent Michaelis-Menten constant of 0.12 mM indicated that the Mb immobilized on the Au-PTy-*f*-MWCNT film retained its native activity. This biosensor can be successfully applied to detect H_2_O_2_ and nitrite in disinfectant cream, eye drops, pickle juice, and milk samples.

Nitrite (NO_2_^−^) contamination is now recognized as a serious hazard to public health, and the concentration of nitrites in groundwater, rivers, lakes, and the environment is increasing^1^. Nitrite is largely used in the production of beverage and food products, and as an important precursor in the formation of N-nitrosamines, which have been shown to have potent carcinogenic effects in humans^2^. Therefore, the measurement of nitrites in specific materials has received a great deal of attention in recent years. Sensitive, selective, and precise methods are required for the determination of nitrite concentrations in a sample^3^. Several analytical technologies have been utilized for nitrite measurement, including chromatography, chemiluminescence, capillary electrophoresis, and spectrophotometry. However, electrochemical-based sensors have the advantages of high sensitivity, relatively good selectivity, and rapid response^4^,^5^. Nitrite sensors have also recently been constructed that utilize immobilized proteins or enzymes in nanomaterials while taking advantage of quantum-size effect and surface effect^6^. The large surface area of nanomaterials provides sufficient active sites to facilitate the immobilization of proteins or enzymes, and provides a satisfactory microenvironment to retain their bioactivity^7^.

Myoglobin (Mb) is a 16.7-kD water-soluble heme-protein with a single polypeptide chain containing an iron heme as its prosthetic group, and it is found in most mammals and vertebrates^8^,^9^. The main physiological function of Mb is oxygen storage to enhance the rate of oxygen diffusion^10^. Mb undergoes a catalytic activity with H_2_O_2_, similar to that of horseradish peroxidase. However, direct electron transfer between the heme-centers of the Mb and traditional bare working electrodes is generally difficult^11^,^12^. Thus, efforts have been devoted to enhancing the electron transfer rate of Mb^13^. Until now, many nanomaterials, such as hydrogel, sol-gel films, carbon nanotubes, gold nanoparticles, and titania-nanotubes, as well as boron-doped diamonds, have been applied to modify the electrode surface^14^,^15^. The properties that make these materials superior are their hydrophilicity, nontoxicity, excellent film-forming ability, and remarkable biocompatibility, which offer excellent prospects for facilitating the direct electrochemistry of redox proteins^16^.

Carbon nanotubes (CNTs) have attracted a great deal of attention in the electroanalysis field due to their unique properties, such as their specific surface area, good mechanical stability, tubular structure, and high electric conductivity^17^,^18^. In addition, the application of multi-walled carbon nanotubes (MWCNTs) has been found to enhance electrocatalytic activity because of the presence of edge-plane-like sites located at both ends and in the defect region^19^,^20^. Tyramine (Ty), 4-(2-aminoethyl) phenol, has also received considerable attention, primarily for use in business and drug-release matrices^21^. Bioelements such as oligonucleotides or enzymes become attached by entrapment, cross-linking, and covalent attachment due to the mild polymerization conditions and the availability of the primary amine group^22^. In recent studies, poly tyramine (PTy) was found to be a biodegradable cationic polymer, and it has attracted a great deal of attention due to its biocompatibility, non-toxicity, low-cost, and good film-forming ability^23^. It is well known that Ty can be used to stabilize AuNPs to form the Ty-AuNP composite, which supports excellent chelating and film-forming ability^24^. Moreover, composite materials possess the properties of each component, making them useful for studies of the direct electron transfer reactions of proteins^25^. However, the design of efficient nitrite sensors based on immobilization of Mb on electrode surfaces modified with novel composites is still a challenge.

In the present study, a flexible, transparent Mb/Au-PTy-*f*-MWCNT composite was fabricated and attached through electrostatic interaction to a glassy carbon electrode (GCE) surface. This composite acts as a robust composite for the immobilization of Mb. Ty is a biopolymer composed of a carbon-chain backbone to which hydroxyl groups are attached. This composite can provide a biocompatible microenvironment for protein or enzyme immobilization that improves the stability of the modified electrode. Mb/Au-PTy-*f*-MWCNT biocomposites are used for H_2_O_2_ and nitrite detection because of their nontoxic properties, high electrocatalytic activity, versatility, and chemical inertness in the presence of oxygen and moisture. Moreover, the results presented herein provide a new method for preparation of a mediator-free biosensor based on Mb/Au-PTy-*f*-MWCNT that is easy and low-cost, and that uses low amounts of solvents and reagents. To the best of our knowledge, this is the first time such an Mb/Au-PTy-*f*-MWCNT has been used to determine the amount of nitrite in pickle juice and milk samples and to detect H_2_O_2_ in disinfectant cream and eye-drop samples.

## Results and Discussion

**Figure 1:**
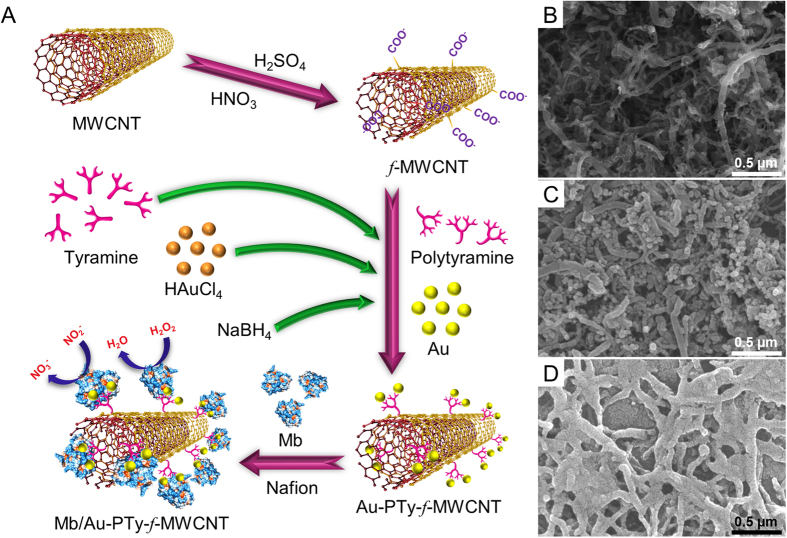
(**A**) Schematic representation of the procedure used to produce the Mb/Au-PTy-*f*-MWCNT/GCE composite, SEM images of (**B**) *f*-MWCNT, (**C**) Au-PTy-*f*-MWCNT, and (**D**) Mb/Au-PTy-*f*-MWCNT biocomposite.

The surface morphology of the *f*-MWCNT, Au-PTy-*f*-MWCNT, Mb/PTy-*f*-MWCNT, and Mb/Au-PTy-*f*-MWCNT biocomposites was investigated by FE-SEM. The electrode surface was mostly covered with homogenous MWCNT in the form of small bundles of tubes (Fig. 1B).

The results shown in Fig. 1C were obtained after mixing the PTy-*f*-MWCNT composite with Au nanoparticles. The Au nanoparticles were clearly visible on the surface of the PTy-*f*-MWCNT composite, indicating that they were bound to the nitrogen groups there. In addition, the PTy-*f*-MWCNT clearly became more corrugated after attachment of the Au nanoparticles. Elemental mapping of C, O, N, and Au nanoparticles using energy dispersive X-ray (EDX) analysis was conducted to investigate the distribution of Au nanoparticles in the PTy-*f*-MWCNT composite (Fig. S1). The EDX images confirmed that the Au-PTy-*f*-MWCNT composite contained Au in addition to the initial C and O. Moreover, the ratio of the contents (O/C) was somewhat lower than that of the *f*-MWCNT (Fig. S2). These findings indicated that the extent of the deoxygenation was dependent on the attached Au nanoparticles. The area of bright contrast correlated with the Au signal map. It is possible that a layer of Au nanoparticles formed on the surface of the PTy-*f*-MWCNT composite, and more likely that very small nanoparticles formed. Well-adhered Mb molecules coated the Mb/PTy-*f*-MWCNT composite surface with the help of binding due to the nitrogen functional groups (Fig. S3). As shown in Fig. 1D, the morphology of the surface changed drastically following the immobilization of Mb on the Au-PTy-*f*-MWCNT composite, due to the interaction between the nanocomposite and the Mb molecules. Moreover, the distribution of Mb molecules throughout the structure of the PTy-*f*-MWCNT composite may have led to the formation of electrostatic interactions between the enzyme and the Au nanoparticles.

**Figure 2:**
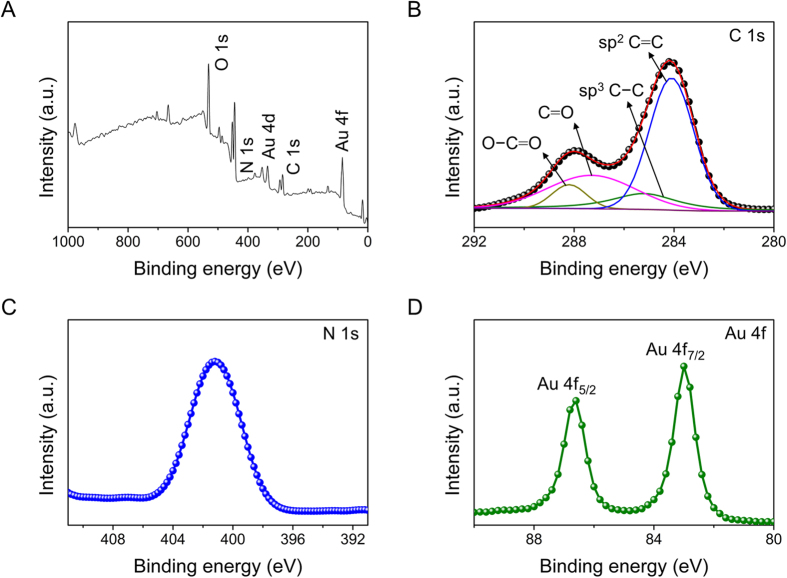
(**A**) XPS survey spectra of the *f*-MWCNTs-PTy-Au composite, (**B**) XPS core level spectra of C 1 s, (**C**) N 1 s, and (**D**) Au 4 f.

The chemical states of the Au-PTy-*f*-MWCNT composites were detected by XPS. As shown in Fig. 2A, there were four dominant elements: C, Au, O, and N. Figure 2B illustrates the core level, the C 1 s region, which shows graphitic C = C bonds (285.2 eV) and different contributions of C−C (285.2 eV), C = O (287.3 eV), and O−C = O bonds (288.2 eV) coming from structural defects, native surface groups, and moisture in the pristine MWCNT sample. The N 1 s spectrum obtained from the Au-PTy-*f*-MWCNT composites were fitted to investigate the chemical bonding structure of the N atoms in *f*-MWCNT (Fig. 2C). The peaks with binding energies at 398.6, 400.0, and 401.7 eV represent pyridinic, pyrrolic, and quaternary nitrogen, respectively. These results demonstrate that substitution of the N atoms occurs at the edge of *f*-MWCNT, and N atoms were successfully incorporated into the *f*-MWCNT surface. Figure 2D displays the typical Au 4f core-level XPS spectrum in which the spin-orbit split between peak positions of the energy band, observed at 83.8 eV (Au 4 f_7/2_) and 87.5 eV (Au 4 f_5/2_), correspond to the Au^(0)^ oxidation state. In addition, there were no observed Au^(III)^ peaks, which are usually observed at a binding energy of around 92 eV, indicating that most of the AuCl_4_^−^ on the sidewalls of the PTy-*f*-MWCNT had been reduced to Au^(0)^. This is indicates the presence of Au in the PTy-*f*-MWCNT composites^26^,^27^.

**Figure 3:**
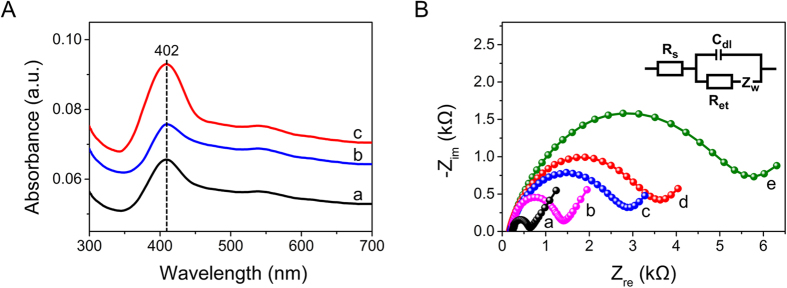
(**A**) UV-vis spectra of the (a) Mb, (b) Mb/PTy-*f*-MWCNT, and (c) Mb/Au-PTy-*f*-MWCNT films. (**B**) EIS results for (a) bare electrode, (b) Au-PTy-*f*-MWCNT/GCE, (c) PTy-*f*-MWCNT/GCE, (d) Mb/Au-PTy-*f*-MWCNT/GCE, and (e) Mb/GCE in the 5 mM Fe(CN)_6_^4−/3−^ containing 0.05 M PBS buffer solution. Inset: Randles equivalent circuit model.

It is well known that UV-Vis spectroscopy is an effective means of determining the characteristic structure of Soret absorption bands for the four iron heme groups in heme proteins, and it may provide information regarding the conformational integrity of the proteins and possible denaturation or conformational changes in the heme region. Dry Mb has a sensitive Soret absorption band at around 402 nm, as shown in Fig. 3A. It is also well known that the PTy-*f*-MWCNT composite forms a suitable medium for the immobilization of Mb onto ITO. The Mb film is negatively charged, and electrostatic interaction with the positive charge of the PTy-*f*-MWCNT composite promotes the composite’s stability. Following immobilization of Mb in the Au-PTy-*f*-MWCNT composite, an absorption peak of 402 nm is obtained. The position of this peak is identical to that of the native Mb film, indicating that the native structure and conformation of the immobilized Mb in the Au-PTy-*f*-MWCNT composite were well-retained. In other words, the Au-PTy-*f*-MWCNT composite provides a microenvironment in which Mb can retain its native structure. These findings suggest that Mb may retain its native structure when it is immobilized in the Mb/Au-PTy-*f*-MWCNT composite^28^,^29^.

Electrochemical impedance spectroscopy (EIS) is an effective tool for probing the interfacial properties of the electrode during the modification process. The Nyquist plot shows a semicircular region at higher frequencies that corresponds to the electron-transfer resistance (R_et_), and a linear region at lower frequencies that corresponds to the diffusion process. The impedance data were fitted to the Randles circuit (see the inset in Fig. 3B). This Randles equivalent circuit contains four circuit elements, including the solution-phase resistance (*Rs*), the charge-transfer resistance (R_et_), the Warburg impedance (*W*), and the double-layer capacitance (*C*_dl_). According to the Nyquist diagram, a semicircular area with an *R*_et_ of about 719.8 Ω was obtained for the bare electrode. After immobilization of PTy with *f*-MWCNTs, the value of *R*_et_ increased to 2926 Ω. The increase in *R*_et_ was due to the immobilization of positively charged PTy-*f*-MWCNT on the substrate surface, which resulted in a negatively charged interface that electrostatically repelled the negatively charged redox probe Fe(CN)_6_^3−/4−^ and inhibited the interfacial charge transfer. Following the immobilization of PTy-*f*-MWCNT with Au particles on the electrode surface, the EIS of the resulting film shows an obvious semicircular domain with an R_et_ diameter of about 1418 Ω. This was due to the acceleration of electron transfer, perhaps in response to promotion of the electron transfer rate between Fe(CN)_6_^3−/4−^ and the GCE surface. After modification of the Mb on the GCE surface, this may have occurred because the Mb immobilized on the GCE slowed down the electron transfer at the redox probe due to the electrostatic repulsion between the negatively charged surface of the Mb electrode and the negatively charged probe in the solution. Therefore, the increase in R_et_ can be attributed to the fact that most biological molecules, including enzymes, are poor electrical conductors at low frequencies, which can hinder electron transfer. Conversely, the value of the R_et_ was 3746 Ω for the Mb/Au-PTy-*f*-MWCNT electrode, which was lower than that for Mb/GCE (R_et_ 5902 Ω). These results also confirm the immobilization of enzyme Mb on the Au-PTy-*f*-MWCNT-modified electrode surface.

**Figure 4:**
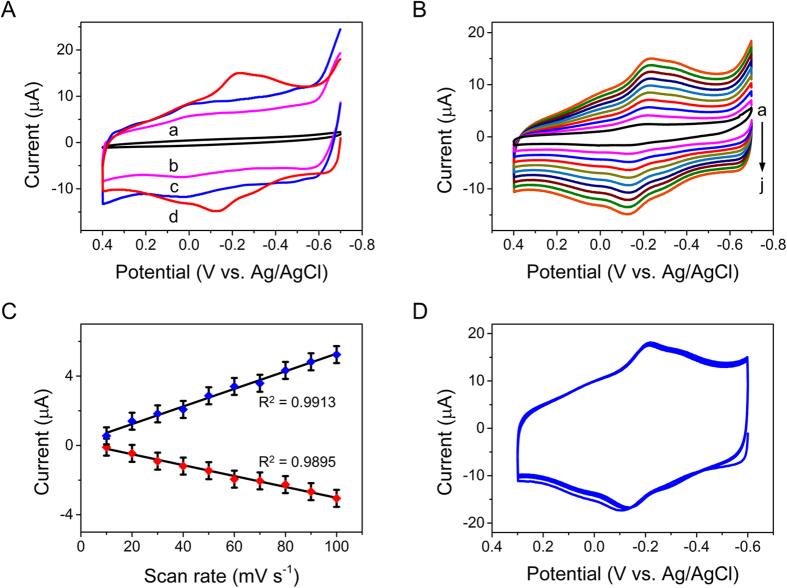
(**A**) CVs of (a) bare electrode, (b) *f*-MWCNT-PTy, (c) Au-PTy-*f*-MWCNT composite, and (d) Mb/Au-PTy-*f*-MWCNT-modified electrodes in 0.05 M deoxygenated PBS (pH 7) at a scan rate of 50 mV s^−1^. (**B**) CVs of Mb/Au-PTy-*f*-MWCNT-modified electrode in N_2_-saturated PBS (0.05 M, pH 6.5) at different scan rates of (a–j) 10, 20, 30, 40, 50, 60, 70, 80, 90, and 100 mV s^−1^. (**C**) Plot of the anodic and cathodic peak currents vs. scan rates. (**D**) Cyclic voltammograms of Mb/Au-PTy-*f*-MWCNT biocomposite-modified electrode for 100 multiple cycles in 10 μM H_2_O_2_ in 0.05 M PBS (pH 7) at a scan rate of 100 mV s^−1^.

To investigate the electrochemical properties of different modified electrodes, (a) bare electrode, (b) PTy-*f*-MWCNT, (c) Au-PTy-*f*-MWCNT, and (d) Mb/Au-PTy-*f*-MWCNT in PBS (pH 7) were recorded (Fig. 4A). In the absence of Mb, no obvious redox peaks were observed in bare GCE or PTy-*f*-MWCNT. Moreover, Mb immobilized on *f*-MWCNT showed no peaks. Conversely, the CVs for Mb/Au-PTy-*f*-MWCNT showed a pair of well-defined redox peaks appearing in Mb, which could be attributed to the direct electron transfer involved in the redox process between Mb-Fe(III) and Mb-Fe(II) with the anodic peak potential (Epa) at −0.147 V and the cathodic peak potential (Epc) at −0.184 V. The peak potential separation (ΔE_p_) was about 37 mV. The results indicate that Mb undergoes a reversible electrochemical reaction and the Mb immobilized on Au-PTy-*f*-MWCNT is not denatured. Moreover, the results indicated that the background current of the Mb/Au-PTy-*f*-MWCNT electrode was higher than that of the bare GCE, PTy-*f*-MWCNT, and Au-PTy-*f*-MWCNT composites. This was attributed to the higher amount of defective sites of *f*-MWCNT, which improved the conductivity of the Au-PTy-*f*-MWCNT composite by forming microchannels beneficial to electron transfer between the modified electrode and the Mb. These results indicate that the Mb/Au-PTy-*f*-MWCNT composite is more beneficial to the direct electrochemistry of Mb than the bare GCE and PTy-*f*-MWCNT composite. Thus, the convenient direct electrochemistry of Mb can be attributed to a synergistic effect with Au-PTy-*f*-MWCNT, which can preserve the biological activity of Mb and provide a favorable microenvironment.

The cyclic voltammograms of Mb/Au-PTy-*f*-MWCNT in pH 7.0 PBS at different scan rates are seen in Fig. 4B. Both the anodic and cathodic peak currents increased the scan rate (ν) and were linear to ν in the range of 0.01–0.1Vs^−1^. The linear regression equations for the cathodic and anodic peaks, I_pc_ = 1.508 + 0.282 (R^2^ = 0.9923) and I_pa_ = −1.034 − 0.122 (R^2^ = 0.9906), indicated a surface-confined electrochemical process. According to the Laviron equation^30^:


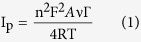


where Г (mol cm^−2^) is the surface amount of Mb adsorbed on the electrode surface, A is the electrode area (cm^2^), ν is the scan rate, I_p_ is the peak current, n is the number of electrons transferred, and F is Faraday’s constant. Therefore, the redox of Mb on Au-PTy-*f*-MWCNT is a single electron transfer reaction. The average surface coverage of Mb on the surface of the modified electrode was estimated to be Γ = 2.12 × 10^−9^ mol cm^−2^, which is much larger than the theoretical monolayer coverage of 1.58 × 10^−11^ mol cm^−2^ estimated for Mb^31^.

The heterogeneous electron transfer rate constant (*k*_s_) between Mb and the Au-PTy-*f*-MWCNT/GCE can be obtained by using the approach developed by Laviron. The relationship between the peak potential (*E*_p_) and the scan rate (where nΔ*E*_p_ > 0.200 V) can be expressed as follows^32^:


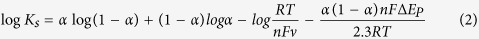


where *α* is the charge transfer coefficient (calculated to be 0.5 using the Tafel equation); *ν* is the scan rate in V s^−1^; *n* is the number of electrons; and *R*, *T*, and *F* are as usually described: R = 8.314 J K^−1^ mol^−1^, T = 298 K, and F = 96,485 C mol^−1^. The *k*_s_ value of the Mb/Au-PTy-*f*-MWCNT was calculated to be 4.86 s^−1^, which is comparatively larger than the values reported in the literature (see Table 1). The higher *k*_s_ value achieved for the Au-PTy-*f*-MWCNT demonstrates the occurrence of rapid electron transfer between the redox active sites of Mb and the modified electrode surface. This is suggestive of the reasonably fast electron transfer between the immobilized Mb and the electrode due to the presence of Au-PTy-*f*-MWCNT. In addition, Fig. 4D illustrates the stability results for the Mb/Au-PTy-*f*-MWCNT-modified GCE. The reduction peak current of the Mb/Au-PTy-*f*-MWCNT-modified GCE in 10 μM H_2_O_2_ was measured for 100 cycles. There was only a 2% decrease in the current density even after 100 cycles, indicating the good stability of the Mb/Au-PTy-*f*-MWCNT nanocomposite-modified electrode surface. The long-term stability and acceptable reproducibility of the biofilm can be attributed to the presence of the Mb/Au-PTy-*f*-MWCNT nanocomposite, which provided a favorable microenvironment for maintaining the bioactivity of the immobilized Mb and for preventing the leakage of Mb.

**Figure 5:**
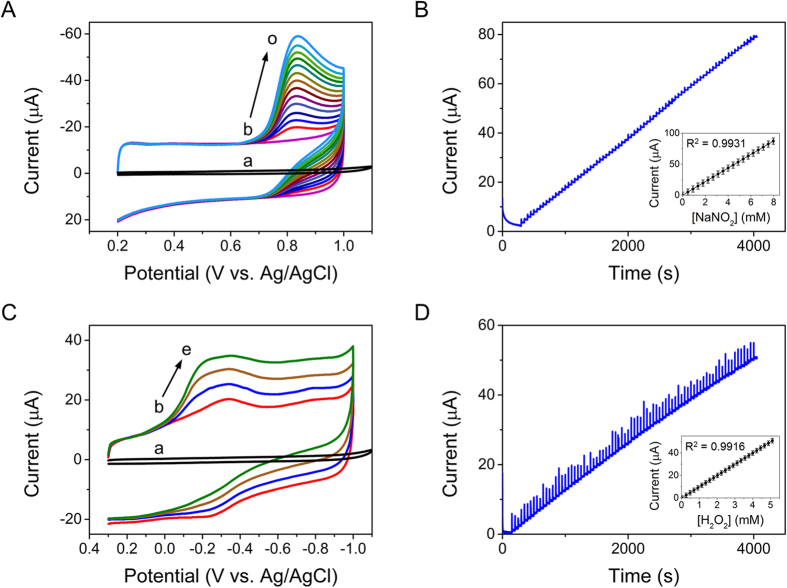
(**A**) CVs of (a) bare electrode and (b) Mb/Au-PTy-*f*-MWCNT/GCE, with added different concentrations of NaNO_2_ (b–o) 0.4–10 mM in pH 7.0 buffer solution (from inner to outer); scan rate: 50 mV/s. (**B**) Amperometric response curves of Mb/Au-PTy-*f*-MWCNT-modified rotating electrode upon successive addition of NaNO_2_ into pH 7.0 PBS solution. Applied potential: +0.74 V. Inset: calibration curve of steady-state currents vs. NaNO_2_ concentration of Mb/Au-PTy-*f*-MWCNT biocomposite. (**C**) CVs of (**a**) bare electrode and (b–e) Mb/Au-PTy-*f*-MWCNT/GCE in PBS free of H_2_O_2_ with various concentrations from 0 to 4 mM in N_2_-saturated PBS (0.05 M, pH 7). (**D**) The amperometric response of the Mb/Au-PTy-*f*-MWCNT-modified rotating electrode for the successive additions of H_2_O_2_ (conditions: −0.3V constant potential, pH 7.0, rotation speed 1200 rpm).

Figure 5A shows the results obtained from the investigation of the CVs of Mb/Au-PTy-*f*-MWCNT in 0.05 M pH 7.0 PBS with different concentrations of NaNO_2_. When NaNO_2_ was added to pH 7.0 PBS, the oxidation peak currents increased obviously as the peak current decreased, indicating a typical electrocatalytic oxidation process that may have been due to the reaction of MbFe(II) with NaNO_2_. Furthermore, the oxidation peak current increased with increasing concentration of NaNO_2_, and the peak current was higher for 0.05 mM NaNO_2_ at *f*-MWCNT-PTy-Au than for the bare GCE. However, this phenomenon was not observed for the bare electrode within the same potential window. These results further confirmed that the Au-PTy-*f*-MWCNT provided a friendly platform for the immobilization of Mb and the bioelectrocatalysis to NaNO_2_. The electrocatalytic process can be expressed as follows^33^:


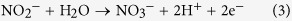


Under optimized conditions, typical amperometric responses of the prepared sensor were recorded following successive additions of NaNO_2_ to 0.05 M pH 7 PBS. Figure 5B shows the amperometric response of the Mb/Au-PTy-*f*-MWCNT at 0.74 V after the successive additions of different concentrations of NaNO_2_. Using the Mb/Au-PTy-*f*-MWCNT electrode, a steady-state current could be obtained in less than 5 s, indicating a very rapid response to changes in the NaNO_2_ concentration. A linear calibration plot was made between the concentration of NaNO_2_ and the peak current. The fabricated biosensor exhibited a wide linear range of 1−8000 μM with a linear regression equation of I(μA) = 12.089 C [NO_2_^−^]/mM + 0.1498 (R^2^ = 0.993). The sensitivity of the biosensor calculated from the slope of the calibration plot was 168 μA mM^−1^ cm^−2^. These findings indicate that the proposed method has the potential to be used for sensitive monitoring of the concentration of NaNO_2_. The limit of detection was found to be 0.002 μM based on LOD = 3S_b_/S, where S_b_ is the standard deviation of 10 blank measurements and S is the sensitivity. The lower detection limit could be ascribed to the high loading of Mb on the Au-PTy-*f*-MWCNT surface, made possible by the present method and the rapid electron transfer between Mb and the electrode. When the concentration of NaNO_2_ was further increased, a current plateau was observed on the calibration curve, which is characteristic of the typical Michaelis-Menten kinetic mechanism. The apparent Michaelis-Menten constant (K_m_), which gives an indication of the enzyme substrate kinetics, can be obtained from the Lineweaver-Burk equation^34^:


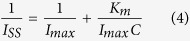


where I_ss_ is the steady-state current after addition of the substrate, *I*_*max*_ is the maximum current measured under saturated substrate conditions, and C is the bulk concentration of the substrate. The linear regression equation is y = 0.426× + 0.098 (R = 0.9932), where y and x are 1/*I*_*ss*_ (mA)^−1^ and *1/C* (mM)^−1^, respectively. K_m_ was obtained by analysis of the slope and intercept of the plot of the reciprocals of the steady-state current versus the NaNO_2_ concentration. The K_m_ of the Mb/Au-PTy-*f*-MWCNT composite was calculated to be 0.38 mM. The low value of K_m_ implied that the entrapped Mb possessed a good affinity to NaNO_2_.

Figure 5C displays the electrocatalytic activity of Mb/Au-PTy-*f*-MWCNT toward the reduction of H_2_O_2_ at a scan rate of 0.05 V/s. As can be seen in comparison with Fig. 5C, there was an increase in the reduction peak at about −0.3 V, which was accompanied by the decrease and disappearance of the oxidation peak for the Mb-immobilized Au-PTy-*f*-MWCNT electrodes after the addition of H_2_O_2_ to the PBS. Conversely, there was a gradual increase in the peak cathodic current as the concentration of H_2_O_2_ increased. Moreover, there was a reduction in the peak oxidation currents for the heme Fe^(III)^/Fe^(II)^ redox that was coupled with an increase in Mb. In blank experiments, no cathodic peak was obtained at the bare electrode without H_2_O_2_. These results indicate typical electrocatalytic activity toward the reduction of H_2_O_2_ and show the successful modification of Mb onto Au-PTy-*f*-MWCNT biocomposites, which resulted in good biocompatibility for maintenance of the biological activity of Mb. It is worth noting that the catalytic effects were better for the Mb/Au-PTy-*f*-MWCNT than for the bare electrode. The results of the reduction of H_2_O_2_ on the Mb/Au-PTy-*f*-MWCNT are shown in Fig. 5C^35^.


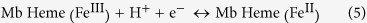



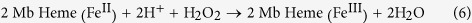


Figure 5D exhibits the amperometric I-t curve, which is the most commonly used method for evaluating the electrocatalytic activity of the Mb/Au-PTy-*f*-MWCNT composite after successive additions of H_2_O_2_ to 0.05 M pH 7 PBS. The results revealed that the optimum applied potential for amperometric determination of H_2_O_2_ was −0.3 V. The electrocatalytic current of the biosensor was observed after each addition of H_2_O_2_ to the solution. The linear range of the curve for the biosensor to H_2_O_2_ concentration was between 2 and 5000 μM with a linear regression equation of I (μA) = 10.092 [H_2_O_2_]/mM + 0.2462 (R = 0.9916). The sensitivity was calculated to be 140 μA mM^−1^ cm^−2^ and the detection limit of the biosensor was 0.01 μM based on a signal-to-noise ratio of 3. These results indicated that the Mb entrapped in the Au-PTy-*f*-MWCNT composite aided in the electrocatalytic activity, which facilitated the measurement of the H_2_O_2_. These results are compared to those reported for other modified electrodes in Table 1. This method was shown to provide a comparable fast electron-transfer-rate constant, linear range and detection limit, stability, and a lower Michaelis-Menten constant value. K_m_ can be observed by an analysis of the slope and the intercept of the plot of the reciprocals of the steady-state current versus the H_2_O_2_ concentration. In this study, the linear equation was expressed as: 1/(*Iss*/μA) = 0.225 + 0.035 1/[*C* (H_2_O_2_)/μM] (R = 0.9914), and the K_m_ value for the electrocatalytic activity of Mb/Au-PTy-*f*-MWCNT to H_2_O_2_ was determined to be 0.12 mM, which implied that the developed electrode exhibited a higher affinity for H_2_O_2_. The low K_m_ value indicated that the Mb immobilized in the Au-PTy-*f*-MWCNT composite retained its bioactivity and had a high biological affinity to H_2_O_2_. Conversely, specificity determination was more important for the biosensor due to interference by coexisting active species, such as ascorbic acid, dopamine, glucose, and uric acid. Therefore, we conducted this study using amperometric measurements. Figure S4 shows the amperometric response of the Mb/Au-PTy-*f*-MWCNT**-**modified electrodes to H_2_O_2_ and different interfering agents, including ascorbic acid, dopamine, glucose, and uric acid. The results revealed no significant interference upon the addition of 100 μM of ascorbic acid (AA), 100 μM of dopamine (DA), 100 μM NaNO_2_, 100 μM of uric acid (UA), or 400 μM of glucose at concentrations 2 times higher than H_2_O_2_ at 50 μM. The above results clearly confirm that the Mb/*f*-MWCNT-PTy-Au biosensor has an acceptable anti-interference ability.

The applicability of the biosensor was investigated to demonstrate its feasibility for practical analysis. The measurement of the proposed sensor was estimated by the standard addition method. The results agree well with those obtained by the conventional UV-vis and titration methods. Samples of a disinfectant cream and eye-drops obtained from a local market were diluted with 0.05 M PBS at a ratio of 1:100 prior to testing, and amperometric measurements were taken after spiking the samples (Table S1). The recovery rates for H_2_O_2_ ranged from 99.2% to 102%. Conversely, the feasibility of usage of the as-prepared Mb/Au-PTy-*f*-MWCNT biosensor to measure the NaNO_2_ in samples of pickle juice and milk was assessed under optimized conditions. Experiments using spiked and recovered samples were conducted with amperometric techniques. The recoveries ranged from 99.1% to 101.4% for NaNO_2_ (Table S1), clearly indicating the applicability and reliability of the proposed method.

The stability and reproducibility rates of the Mb/Au-PTy-*f*-MWCNT biosensor were analyzed. The stability of the Mb/Au-PTy-*f*-MWCNT composite was evaluated after storage in the refrigerator at 4 °C for 2 months. The results revealed that the initial response time decreased by only 5.2%, indicating good stability of the Mb/Au-PTy-*f*-MWCNT composite. Furthermore, the 10 independently fabricated Mb/Au-PTy-*f*-MWCNT composites were tested, and the results showed acceptable reproducibility with a relative standard deviation for the determination of 0.2 mM NaNO_2_ of 2.5%. In general, the Mb/Au-PTy-*f*-MWCNT biocomposites had good repeatability and stability for electrochemical detection. The repeatability of the Mb/Au-PTy-*f*-MWCNT biocomposites biosensor was also investigated and the relative standard deviation was found to be 3.2% (n = 5) for 0.2 mM of NaNO_2_. The good storage stability was ascribed to the high biocompatibility of the Mb/Au-PTy-*f*-MWCNT composite.

## Conclusion

The preparation was further employed for the immobilization of Mb on an Au-PTy-*f*-MWCNT biocomposite surface with a Nafion film. The UV-Vis absorption and EIS results indicated that the Mb retained its native structure in the Au-PTy-*f*-MWCNT biocomposite. Mb was immobilized on the Au-PTy-*f*-MWCNT biocomposite and exhibited reversible, surface-controlled electron-transfer kinetics. Moreover, the method used to prepare this biosensor had some distinct advantages when compared to other complicated immobilization procedures. Specifically, it was simple to construct and easy to operate, and did not require specific reagents. Additionally, the developed electrode was easy to prepare and modify, and facilitated biocatalysis. Accordingly, the methods described herein will be useful for fabricating electrochemical sensors, biosensors, and microelectronics devices, as well as in electrocatalytic processes.

## Methods

### Chemicals

The bovine myoglobin (Mb, MW = 17,800) used in the experiments was purchased from Sigma-Aldrich, the hydrogen peroxide (30%, w/v solution) was obtained from the Beijing Chemical Reagent Company, and the Nafion (5 wt. %), and K_3_Fe(CN) _6_, K_4_Fe(CN) _6_, an_2_HPO_4_, NaH_2_PO_4_, NaNO_2_, and hydrogen tetrachloroaurate hydrate (HAuCl_4_·3H_2_O) were all acquired from Sigma-Aldrich. All chemicals were of analytical reagent grade and were used without further purification. NaNO_2_ and H_2_O_2_ solutions were prepared immediately before use. In addition, 0.05 M phosphate-buffered saline (PBS) solutions with different pH values were prepared by mixing stock solutions of Na_2_HPO_4_ and NaH_2_PO_4_ and then adjusting them by the addition of NaOH and H_2_SO_4_ solutions. Double-distilled water was used throughout the experiments.

### Characterization

Electrochemical measurements were performed using a CHI 405A (CH Instruments, Chenhua, Shanghai, China) electrochemical work station with a conventional three-electrode system comprised of platinum wire as the auxiliary electrode, an Ag/AgCl electrode as the reference electrode, and a modified GCE as the working electrode. All potentials were reported in this context with respect to this reference. All measurements were performed at room temperature (~25 °C). The UV-Vis spectra were recorded on a Model UV-3300 spectrophotometer (Hitachi, Japan). The surface morphology of the modified samples was observed with a scanning electron microscope (SEM) using a Hitachi S-3000 H instrument (Hitachi, Japan) at an accelerating voltage of 15 kV, while EDX was carried out using a Horiba EMAX X-ACT (Model 51-ADD0009). X-ray photoelectron spectroscopy (XPS) was performed using a PHI 5000 Versa Probe equipped with an Al Kalpha X-ray source (1486.6 eV), while EIS measurements were carried out at frequencies ranging from 0.1 Hz to 1 MHz (IM6ex ZAHNER Kroanch, Germany).

### Synthesis of Au-PTy-*f*-MWCNT composite

MWCNT (1 mg) was chemically shortened by ultrasonic agitation in a mixture of sulfuric acid and nitric acid (3:1) for 2 h to obtain a homogeneous mixture. After sonication, the mixture was refluxed for 12 h at 90 °C to obtain the carboxyl functional group (*f*-MWCNT). The mixture was then filtered under vacuum through a 0.45 μm Millipore polycarbonate membrane. The resulting *f*-MWCNT was separated and washed repeatedly with distilled water by centrifugation until the pH was 7. In addition, the acid-treated *f*-MWCNT (10 mg) was dispersed into a 0.5 ml aqueous solution of 2 mg tyramine, after which the resulting dispersion was sonicated for 3 h to produce a homogeneous black suspension. Next, 2 mg of PTy-*f*-MWCNT composite was added to 8 ml of the black suspension and 3 ml of HAuCl_4_ solution (1 wt), and then mixed together in a round-bottom flask. Following mixing, 5 mL of 0.02 mol/L NaBH_4_ was added to the mixture and the solution was subjected to vigorous stirring at 80 °C. The reaction was allowed to continue for another 24 h, after which the sample was allowed to cool to room temperature. Next, the black solid was separated and washed with distilled water several times, then dried at 80 °C. Finally, the Au-PTy-*f*-MWCNT (1 mg ml^−1^) was dispersed in water and adsorbed onto the GCE surface in the Au-PTy-*f*-MWCNT composite-modification process.

### Fabrication of the Mb/Au-PTy-*f*-MWCNT-modified electrode

The sequential biosensor was fabricated on a GCE with a diameter of 3 mm. The electrode was then polished to a mirror finish using a 0.05 μm alumina slurry, after which it was sonicated in nitric acid (1:1), ethanol, and deionized water. Next, the electrode was rinsed with ultra-pure water and allowed to dry under N_2_. The modification procedure is illustrated in Fig. 1A. After modification, 5 μl of the Au-PTy-*f*-MWCNT suspension was dropped onto the pretreated GCE before being dried in silica gel desiccators. The Mb solution was first prepared by adding 10 mg/ml of Mb to 0.05 M pH 7.0 PBS. Next, 5 μL of Mb solution was applied to the surface of the freshly prepared Au-PTy-*f*-MWCNT/GCE, then evaporated at 4 °C in a refrigerator to form a stable film. The electrode was then rinsed with double-distilled water two or three times to remove any loosely bound Mb molecules, and then allowed to dry at ambient temperature overnight. Finally, 1 μL of Nafion (0.1%) was dropped onto the Mb/Au-PTy-*f*-MWCNT film to serve as a binder to hold the composite film onto the electrode surface. The solvent was allowed to evaporate to produce the final Nafion/Mb/Au-PTy-*f*-MWCNT/GCE. When not in use, the enzyme electrode was stored in 0.05 M pH 7.0 PBS at 4 °C. The electrode was also stored in the same PBS overnight and before electrochemical experiments.

## Additional Information

**How to cite this article**: Vilian, A.T. E. *et al.* Immobilization of myoglobin on Au nanoparticles− decorated carbon nanotube/polytyramine composite as a mediator-free H2O2 and nitrite biosensor. *Sci. Rep.* **5**, 18390; doi: 10.1038/srep18390 (2015).

## Supplementary Material

Supplementary Information

## Figures and Tables

**Table 1:** Comparison of the analytical performance of the H_2_O_2_ and NO_2_^−^ over various modified electrodes.

Modified Material Electrode	Analytical methods	Sensor	Γ(mol cm^−2^)	ks (s^−1^)	Applied Potential (V)	Linear range (μM)	LOD (μM)	K_M_(mM)	Ref
Mb-Au/Pyrolytic graphite	Amperometry	H_2_O_2_	2.33 × 10^−10^	—	−0.1 (SCE)	2–80	1.2	—	11
Mb- titanium carbide nanoparticles- Chitosan/GCE	Amperometry	H_2_O_2_	5.86 × 10 ^− 10^	3.8	−0.3 (SCE)	0.5–50	0.20	0.07	12
Gold nanorods@SiO_2_-Mb/room temperature ionic liquid-sol-gel/GCE	CV	H_2_O_2_	7.65 × 10^−9^	4.7	(SCE)	0.2–80	0.12	0.42	14
Nafion/*f*-MWCNTs/MB/Carbon ionic liquid	CV	H_2_O_2_	4.64 × 10 ^−9^	0.332	(SCE)	8–196	6.00	0.0001	15
Chitosan-MWNTs/Mb/AgNPs/GCE	Amperometry	H_2_O_2_	4.16 × 10 ^−9^	5.47	−0.3 (Ag/AgCl)	25–200	1.02	0.024	16
Mb/ZrO_2_/MWCNT/GCE	Amperometry	H_2_O_2_	1.36 × 10^−10^	1.52	−0.4 (Ag/AgCl)	1.00–116	0.53	0.085	19
Clay-ionic liquid (1-butyl-3-methyl imidazolium tetrafluoraborate/Mb/GCE	Amperometry	H_2_O_2_	4.90 × 10^−1^	3.58	−0.15 (Ag/AgCl)	3.9–259	0.73	0.0176	36
Mb/DNA/N-butylpyridinium hexafluorophosphate (BPPF_6_)/Carbon ionic liquid	CV	H_2_O_2_	—	1.02	(SCE)	1.0–160	0.2	0.42	37
Nafion/Mb/ionic liquid/GCE	Amperometry	H_2_O_2_	5.89 × 10^−11^	—	−0.45 (Ag/AgCl)	1.0–180	0.14	0.022	38
Mb/1-butyl pyridinium hexaflourophosphate/Carbon ionic liquid	Amperometry	H_2_O_2_	1.06 × 10^−9^	2.8	−0.39 (SCE)	6.0–160	2	1.40	39
Nafion/MB/colloidal gold nanoparticle/GCE	Amperometry	H_2_O_2_	—	—	−0.45 (SCE)	1.5–90	0.50	—	40
Mb-CeO_2_/Indium tin oxide	Amperometry	H_2_O_2_	5.142 × 10^−11^	1.57	−0.3 (Ag/AgCl)	200–5000	0.6	3.15	41
Mb-1-butyl-3-methyl-imidazolium tetrafluoroborate- hyaluronic acid/GCE	CV	H_2_O_2_	9.56 × 10 ^−11^	4.21	(SCE)	2.0–270	0.6	0.29	42
Nafion/Mb- Poly(methacrylic acid-co-acrylamide)- *f*-MWCNTs/Au	Amperometry	H_2_O_2_	6.3 × 10^−10^	1.644	−0.45 (SCE)	1.47–4760	0.76	—	43
Mb-Dodecyltrimethylammoniumbromide/Carbonceramic	CV	H_2_O_2_	—	3.03	(SCE)	110–1600	40.0	—	44
Mb/Au-PTy-*f*-MWCNT/GCE	Amperometric	H_2_O_2_	2.12 × 10^−9^	4.86	−0.3 (Ag/AgCl)	1–5000	0.01	0.12	This work
Nafion/grapheme/Mb/GCE	CV	NO_2_^−^	—	3.9	—	50–2500	10	—	2
Mb/LaF_3_-DP-CeO_2_/IL/Carbon paste	Amperometric	NO_2_^−^	2.07 × 10^−9^	1.01	0.8 (Ag/AgCl)	5–4650	2.0	2.19	29
Mb/multi-walled carbon nanotube (MWCNT) -cysteamine – Nafion/Au	Amperometric	NO_2_^−^	—	—	0.7 (Ag/AgCl)	1–250	0.1	—	35
Hemoglobin/colloidal Au nanoparticles/TiO_2_/GCE	Amperometric	NO_2_^−^	—	—	−0.75 (Ag/AgCI)	4.0–3500	1.2	—	45
Cytochrome c/DNA/MWCNT- poly(amidoamine)-Chitosan/GCE	Amperometry	NO_2_^−^	8 × 10^−10^	1.5	+0.95 (SCE)	0.2–80	0.03	—	46
Cytochrome c/l-cysteine /poly-3-methylthiophene/multi-walled carbon nanotubes/GCE	Amperometric	NO_2_^−^	1.6 × 10^−11^	0.49	+0.9 (Ag/AgCl)	10–100	0.5	—	47
Hb-ZnO-Nafion/GCE	Amperometric	NO_2_^−^	1.0 × 10^−10^	3.2	−0.675 (Ag/AgCl)	10–2700	4.0	—	48
Nafion-BMIMPF_6_/Mb/Carbon ionic liquid	CV	NO_2_^−^	4.97 × 10^−9^	0.532	(SCE)	100–8400	50	1.46	49
Mb/Au-PTy-*f*-MWCNT/GCE	Amperometric	NO_2_^−^	2.12 × 10^−9^	4.86	+0.74 (Ag/AgCl)	1–8000	0.002	0.38	This work
