# Supplementary material for: Immobilization of myoglobin on Au nanoparticle-decorated carbon nanotube/polytyramine composite as a mediator-free H2O2 and nitrite biosensor
Source: Sci Rep. 2015 Dec 17;5:18390. doi: 10.1038/srep18390 (PMC4682093; doi:10.1038/srep18390)
Supplement: Supplementary Information [file srep18390-s1.pdf]

Supplementary Information

Immobilization of myoglobin on Au nanoparticle-decorated carbon nanotube/polytyramine composite as a mediator-free H_2O_2 and nitrite biosensor

A.T. Ezhil Vilian [1,2], Vediyappan Veeramani[1], Shen-Ming Chen[1,*], Rajesh Madhu[1], Cheol Hwan Kwak[2], Yun Suk Huh [2,*] and Young-Kyu Han [3,*]

[1]Electroanalysis and Bioelectrochemistry Lab, Department of Chemical Engineering and Biotechnology, National Taipei University of Technology, No. 1, Section 3, Chung-Hsiao East Road, Taipei 106, Taiwan, ROC.

[2]Department of Biological Engineering, Biohybrid Systems Research Center (BSRC), Inha University, Incheon 402-751, Republic of Korea.

[3]Department of Energy and Materials Engineering, Dongguk University-Seoul, Seoul 100-715, Republic of Korea.

E-mail: smchen78@ms15.hinet.net (S.M.C) & ykenergy@dongguk.edu (Y.K.H.) & yunsuk.huh@inha.ac.kr (Y.S.H.)

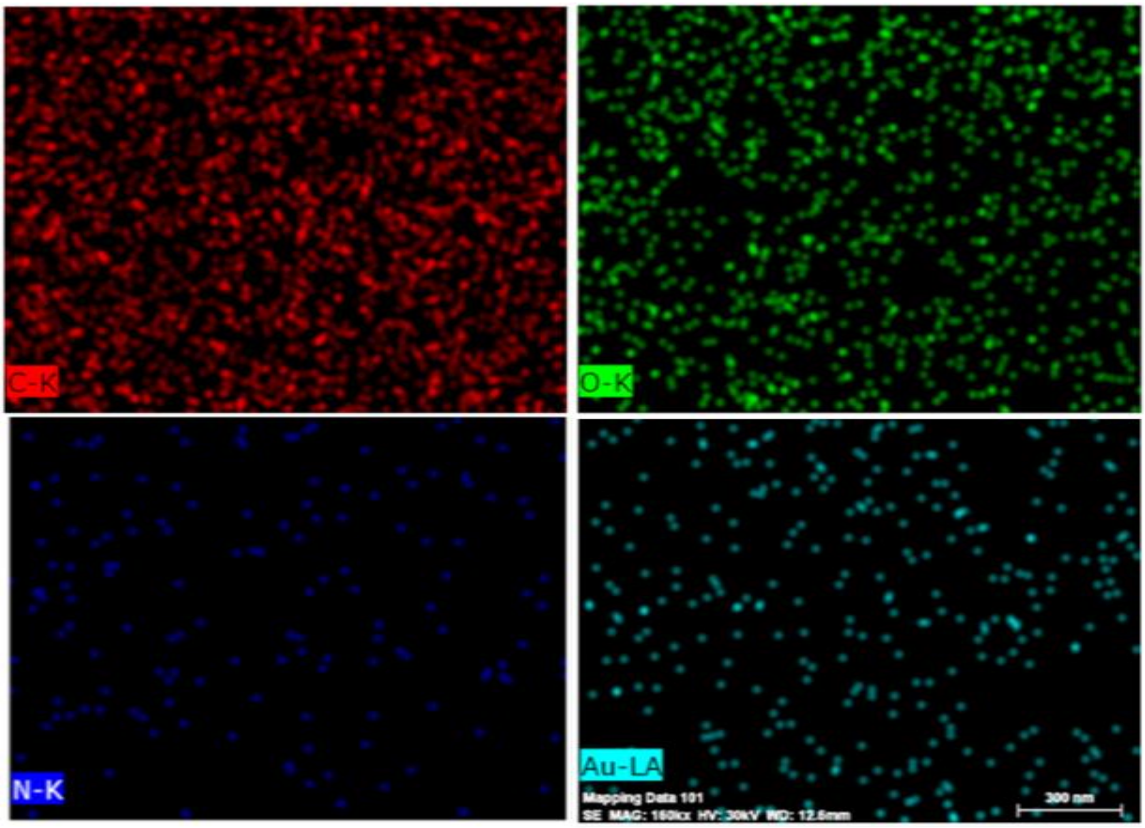

Figure S1. EDX areal maps of (C, O, N, Au) Au-PTy-*f*-MWCNT composite

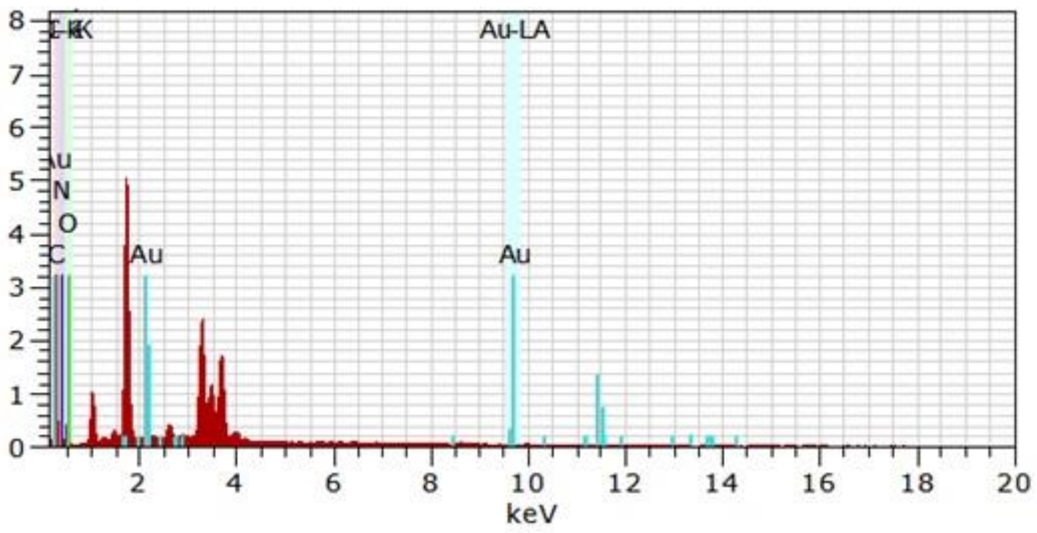

Figure S2. shows the EDX spectra of Au-PTy-*f*-MWCNT /ITO composite films

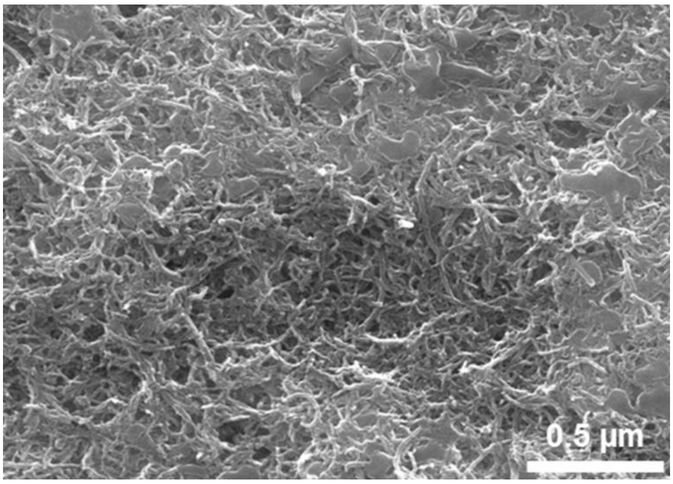

Figure S3. SEM study of Mb coated Pty-*f*-MWCNT composite

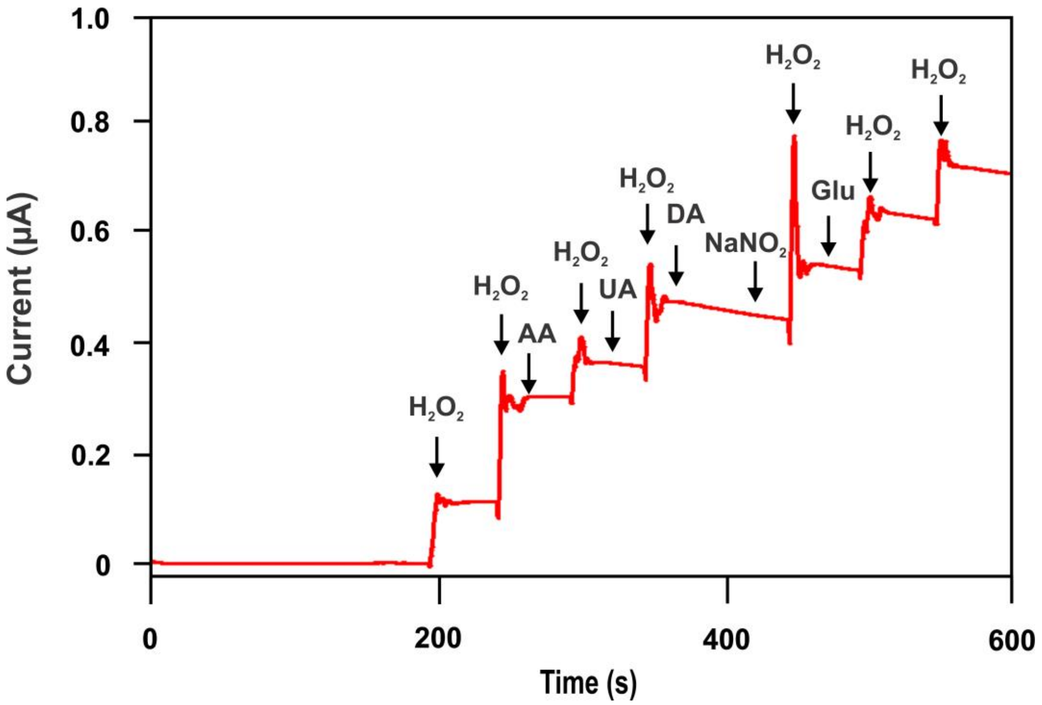

Figure S4. Amperometric i-t response at Mb/Au-PTy-*f*-MWCNT Biocomposite modified rotating disc GCE upon addition of 100 µM of H_2O_2, 100 µM ascorbic acid, 100 µM uric acid, 100 µM dopamine, 100 µM $NaNO_2$ and 100 µM glucose solutions into continuously stirred N_2 saturated PBS. Constant potential: -0.3 V.

Table S1. Determination of H_2O_2 and $NaNO_2$ in real samples obtained using the Mb/Au-PTy-*f*-MWCNT modified electrode

**$NaNO_2$ samples**	**UV-vis method[μM]**	**This work [μM]**	**Added [μM]**	**Found [μM]**	**RSD (%)**	**Recovery (%)**
Milk	80.1	79.4	5	4.8	3.2	100.5%
	81.6	79.8	10	8.9	2.2	99.2%
Pickle juice	79.4	79.1	5	4.6	3.6	102%
	80.2	79.5	10	9.2	2.7	101%
**H_2O_2 samples**	**$KMnO_4$ titration method [μM]**	**This work [μM]**	**Added [μM]**	**Found [μM]**	**RSD (%)**	**Recovery (%)**
Disinfectant cream	40.2	39.6	10	9.4	2.5	99.6
	41.6	39.2	20	19.2	3.2	99.1
Eye drop	39.8	38.4	10	9.6	2.4	100.6
	40.4	39.8	20	19.8	2.9	101.4
